# Correlation analysis between vitamin A, D, and E status with altitude, seasonal variation, and other factors, among children aged 0–6 years in a Chinese population living in the Tibetan plateau of Ganzi prefecture

**DOI:** 10.1002/jcla.24620

**Published:** 2022-07-31

**Authors:** Ping Huang, Gang Ke, Xinmei Lin, Quansheng Wang, Wei Lu, Li Zeng, Shiying Xu

**Affiliations:** ^1^ Department of Child Healthcare Luzhou people's Hospital Luzhou China; ^2^ Department of Pharmacy Luzhou people's Hospital Luzhou China; ^3^ Department of Blood Transfusion Luzhou people's Hospital Luzhou China; ^4^ Department of Rehabilitation Luxian Hospital of TCM Luzhou China; ^5^ Department of Rehabilitation Luzhou People's Hospital Luzhou China; ^6^ Department of Emergency Luzhou People's Hospital Luzhou China

**Keywords:** 25‐hydroxy vitamin D, altitude, plateau Tibetans, seasonal variation, vitamin a, vitamin E

## Abstract

**Objective:**

We attempted to understand the status of vitamin (Vit) A, D, and E in children aged 0–6 living in the Tibetan plateau areas of Ganzi prefecture, to provide the basis for relevant government departments to carry out physical examinations of these children and to prevent and cure four key diseases (Infantile diarrhea, nutritional anemia, rickets, and infantile pneumonia).

**Methods:**

Serum retinol and tocopherol levels were detected using high‐performance liquid chromatography (HPLC). Serum levels of 25‐(OH)D were detected by high‐performance liquid chromatography–tandem mass spectrometry (LC–MS). The polynomial logistic regression was used to analyze the effects of age, season, altitude, and gender on Vit A, D, and E levels.

**Results:**

Vit A and D had the lowest mean serum levels before the age of 1 year and with the most significant deficiency rates. The lowest Vit E levels were seen in the Toddlerhood group. The rates of deficiency and insufficiency were the highest. Vit A, D, and E levels were significantly affected by seasonal changes and were significantly higher in the summer than in any other season. Vit A and D were significantly affected by altitude, and their levels were lowest above 4 km.

**Conclusion:**

The overall levels of Vit A, D, and E in children aged 0–6 in the Tibetan plateau areas of Ganzi prefecture were lower than those in the plain's areas.

## Graphical abstract

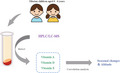

## 1 INTRODUCTION

Vitamins are essential regulatory amines for the maintenance of normal health and play an important role in cellular metabolism. Because of rapid economic development and changes in children's diet, activity, and lifestyle, the medical community has become involved in the search for a relationship between vitamin deficiency and their health and disease status. Therefore, it is of great importance to prevent vitamin deficiency in children, and vitamin (Vit) A deficiency has been recognized by the World Health Organization (WHO) as one of the four major nutritional deficiencies worldwide.^1^ Mild Vit A deficiency may also lead to decreased immune function and increased respiratory and digestive infections in children.^2^ At present, the nutritional status of Vit A in Chinese children has been significantly improved,^3^ Vit A deficiency with its typical associated clinical manifestations is relatively rare, but subclinical and marginal subclinical Vit A deficiency cannot be ignored.^3^ Vit A deficiency remains a moderate public health problem for school‐age children.^4^, ^5^, ^6^ This vitamin is important for child health, and its deficiency not only affects existing health but also extends to long‐term health in adulthood.^7^ The prevalence of Vit D deficiency is high among children aged 0–6 years in China, especially among preschoolers.^8^ Vit E can inhibit the formation of free radicals and can maintain the stability of cell membranes and enhance the immune system. In addition to influencing the level of immunoglobulins, Vits E and A can synergistically influence immune function.^9^

The WHO defines Vit A deficiency (VAD) as tissue concentrations of the vitamin low enough to have adverse health consequences, even if there is no evidence of clinical deficiency.^10^ The serum retinol level is a reliable indicator of the Vit A status of an individual.^11^ According to the WHO and the Pediatrics branch of The Chinese Medical Association, serum retinol levels SRLs are classified as ≥1.05 μmol/L for normal, 0.70–1.05 μmol/L for marginal VAD (MVAD), and <0.70 μmol/L defines full blown VAD.^12^ SRLs between 0.35 and 0.7 μmol/L are considered subclinical Vit A deficiency (SVAD), whereas a level of ≤0.35 μmol/L is considered to be clinically deficient.

The principle stored form of Vit D in the body is 25‐(OH) D and is therefore used as a gold standard index for the evaluation of the nutritional status of Vit D.^13^, ^14^ In 2007, Chinese Pediatric Society Chinese Medical Association stated that Vit D deficiency is defined as a serum 25(OH)D level of <37.5 nmol/L and Vit D insufficiency as a serum 25(OH)D level of 37.5–50 nmol/L.^15^ However, controversy remains concerning the correct healthy level of 25‐hydroxyvitamin D (25[OH]D) in children and even what level of 25(OH)D should be used to define Vit D deficiency. Although controversial,^16^, ^17^ higher levels of 25(OH)D have been shown in some studies, to improve bone health and may also help prevent cancer, autoimmune diseases/type 1 diabetes, some infectious diseases, and childhood wheezing.^18^, ^19^, ^20^, ^21^, ^22^ In a 2018 consensus written on behalf of the three Italy pediatric‐related associations, it reviewed the definition of vitamin D status according to several societies and organizations in the last 10 years. It was stated that serum 25(OH)D levels of <25, <50, and <75 nmol/L as cutoff points for the definition of vitamin D status severe deficiency, deficiency, and insufficiency.^23^

α‐tocopherol is the most biologically active form of Vit E and children with serum α‐tocopherol concentrations below 11.6 μmol/L (5 mg/L) are considered to be Vit E deficient.^24^, ^25^ As found in pediatric textbooks in China, α‐tocopherol concentrations below 11.6 μmol/L are defined as Vit E deficient, 11.6–16.24 μmol/L as Vit E insufficiency, and ≥16.24 μmol/L as Vit E normal.

Vits A, D, and E play key roles in the support of life. In China, there have been many reports concerning vitamin levels in children, especially Vits A and D,^26^, ^27^ but most of these studies were regional surveys, and there have been no reports on ethnic minorities in plateau areas. Therefore, the purpose of this study was to evaluate Vit A, D, and E status among children aged 0–6 years of age in Ganzi Prefecture, Sichuan Province, China. And investigate vitamin levels and their relationship to other factors, including gender, age, seasonal change, and altitude.

## 2 METHODS

### 2.1 Participants

From April 2017 to April 2019, participants (Tibetan children aged 0–6 years) from Daocheng county and Xiangcheng County, Ganzi Prefecture, Sichuan Province, China, were involved in a comprehensive health checkup. This was approved by the ethics committee of the Luzhou People's Hospital. The parents or guardians of the participants all agreed to this study. Initially, a total of 2650 children aged 0–6 years were recruited, and the following conditions were excluded: metabolic diseases, scoliosis, tuberculosis, kidney disease, and other underlying diseases. After removing those who did not agree with the informed consent, we were left with a total of 1815.

Two milliliters of venous blood was collected into anticoagulation vacuum vessels, and the serum was separated, frozen, and stored in the dark until use. The concentrations of serum retinol and α‐tocopherol were determined by HPLC (LC‐20 High Performance Liquid chromatograph, Shimadzu, Japan). 25‐(OH)D was determined by LC–MS (LC–MS 8040 high‐performance liquid chromatography plain combination instrument, Shimadzu, Japan) as described previously.^28^, ^29^ All standard reagents were purchased from Sigma‐Aldrich, USA. The inter‐assay and intra‐assay coefficients of variation were <5% and <10%.

### 2.2 Statistical analyses

SPSS 22.0 statistical software was used for data analysis and to analyze the correlations between Vit A, 25‐(OH)D and Vit E levels and child age, season, altitude, and gender. Each group generally adopted a single factor analysis of variance, Inter‐group comparisons were performed using 2‐way ANOVA followed by the Duncan multirange test. And a chi‐squared test was used for comparison between groups. Polynomial logistic regression was used to analyze the effects of age, season, altitude, and gender on Vit A, D, and E levels. A two‐tailed *p* value of less than .05 was considered statistically significant. The season during which each serum sample was tacked was classified as spring (March through May), summer (June through August), autumn (September through November), and winter (December through February). The children were divided into groups depending upon age as follows: Infant group was defined as the stage of life from birth to 1 year old (0–1 years of age), the toddler group was defined as the age group between 1 and 3 years old (1–3 years of age), the preschool group was defined as the age group between 3 and 6 years old (3–6 years of age). The altitude was divided into 2–3 km, and 3–4 km or higher.

## 3 RESULTS

### 3.1 Overall levels and prevalence of Vit A, D and E

In this survey, as the results are shown in Table 1, the mean serum retinol for the 1815 Tibetan children aged 0–6 years in Ganzi Prefecture in China was 1.06 ± 0.27 μmol/L, and 8.04% of the children were in VAD. Most of the serum retinol concentrations of these children were between 0.35 and 0.70 μmol/L, and only three children were <0.35 μmol/L. No children presented with typical clinical manifestations of Vit A deficiency, such as night blindness, corneal dryness, ulcers, or Bitt's spot. The mean serum retinol in the infant group was 0.94 ± 0.26 μmol/L, which was lower than that in the toddler group (1.09 ± 0.28 μmol/L) or the preschool group (1.06 ± 0.27 μmol/L). The mean serum retinol in spring was 1.00 ± 0.25 μmol/L, which was lower than that in summer (1.24 ± 0.26 μmol/L), autumn (1.06 ± 0.28 μmol/L), and winter (1.14 ± 0.30 μmol/L). At the same time, the mean serum retinol of children living above 4 km was 0.96 ± 0.19 μmol/L, which was lower than that of children living above 2–3 km (1.12 ± 0.28 μmol/L) and 3–4 km (1.00 ± 0.26 μmol/L). One‐way ANOVA showed differences in age, season, and altitude, whereas the *t* test showed no difference in serum retinol levels between gender (*p* = .279). The incidence of VAD (8.04%) and MVAD (45.29%) in these children was relatively high and when broken down, the incidence of VAD in the infant group was 23.33%, higher than that in the toddler group (6.28%), preschool group (7.21%), or in the overall incidence (8.04%). The incidence of VAD was 10.97% and 9.86% in children living at altitudes of 3–4 km and above 4 km, which was significantly higher than those children living at altitudes of 2–3 km (5.13%), the incidence of MVAD was the same.

**TABLE 1:** Groups of vitamin A concentrations stratified by age, season, and gender

Variables	Number	Mean (μmol/L)	*p* value	Vitamin A Status			*p* value
				<0.70 μmol/L	0.70–1.05 μmol/L	>1.05 μmol/L	
				*n* (%)	*n* (%)	*n* (%)	
Total population	1815	1.06 ± 0.27		146 (8.04)	822 (45.29)	847 (46.67)	
Age group			<.001				<.001
Infant group	120	0.94 ± 0.26		28 (23.33)	49 (40.83)	43 (35.83)	
Toddlerhood group	446	1.09 ± 0.28		28 (6.28)	182 (40.81)	236 (52.91)	
Preschool group	1249	1.06 ± 0.27		90 (7.21)	591 (47.32)	568 (45.48)	
Season			<.001				<.001
Spring	852	1.00 ± 0.25		89 (10.45)	422 (49.53)	341 (40.02)	
Summer	187	1.24 ± 0.26		3 (1.60)	41 (21.93)	143 (76.47)	
Autumn	651	1.06 ± 0.28		50 (7.68)	311 (47.77)	290 (44.55)	
Winter	125	1.14 ± 0.30		4 (3.20)	48 (38.40)	73 (58.40)	
Elevation			<.001				<.001
2–3 km	896	1.12 ± 0.28		46 (5.13)	335 (37.39)	515 (57.48)	
3–4 km	848	1.00 ± 0.26		93 (10.97)	443 (52.24)	312 (36.79)	
>4 km	71	0.96 ± 0.19		7 (9.86)	44 (61.97)	20 (28.17)	
Gender			.279				.002
Boy	885	1.05 ± 0.27		77 (8.70)	400 (45.20)	408 (46.10)	
Girl	930	1.06 ± 0.28		69 (7.42)	422 (45.38)	439 (47.20)	

**FIGURE 1:**
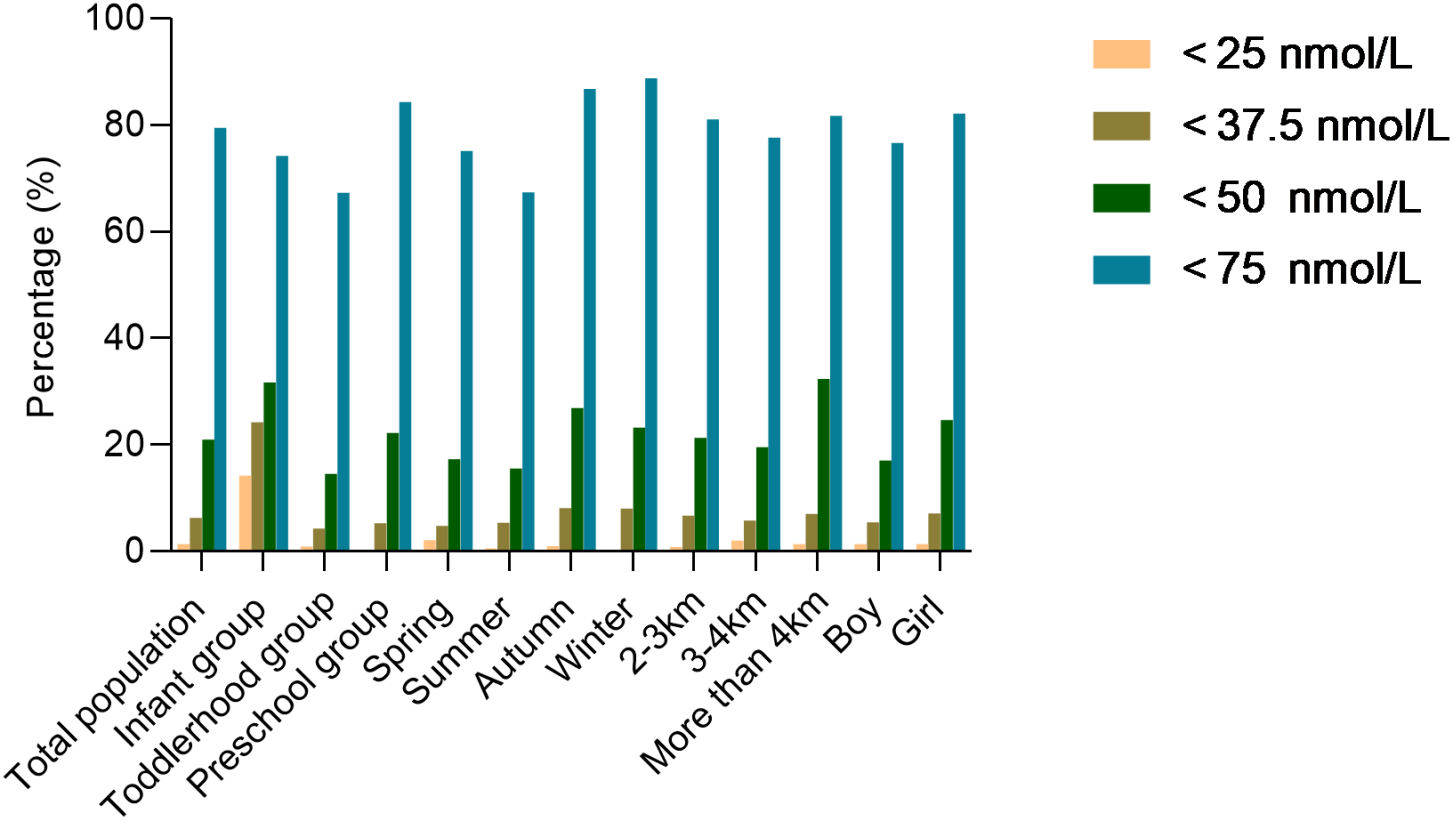
Vitamin D deficiency rate of different standards

As shown in Table 2, the mean serum 25(OH)D was 62.29 ± 16.43 nmol/L, and the mean serum 25(OH)D in the infant group was 57.55 ± 27.11 nmol/L, which was lower than that in the toddler group (67.40 ± 17.03 nmol/L) and the preschool group (60.92 ± 14.34 nmol/L). The mean serum 25(OH)D in summer was (67.53 ± 18.34 nmol/L), which was higher than that in spring (64.37 ± 16.87 nmol/L), autumn (58.61 ± 14.64 nmol/L), and winter (59.39 ± 14.81 nmol/L). The mean serum 25(OH)D within the group living above 4 km was (58.99 ± 18.41 nmol/L), which was lower than that of the group living above 2–3 km (61.76 ± 15.75 nmol/L) and 3–4 km (63.13 ± 16.92 nmol/L). One‐way ANOVA showed differences in age and seasonal elevation, while the *t* test showed no difference in serum 25(OH)D between gender (*p* = .859). Figure 1. shows the prevalence of serum 25(OH)D levels for the children as a group at three thresholds: <25, <50, and <75 nmol/L. Although the prevalence of children aged 1–6 years with a serum 25(OH)D level of <25 nmol/L was not high (1.38%), the infant group (14.17%), clearly had the highest percentage. When we used the definition of deficiency (serum 25(OH)D ≤ 37.5 nmol/L),^15^ the prevalence of Vit D deficiency increased to 6.28% for all the children aged 1–6 years. In the infant group, the deficiency rate increased to 24.17%. When a threshold of <50 nmol/L was used, approximately one‐fifth of the children had low levels of serum 25(OH)D. Furthermore, if a serum level of <75 nmol/L 25(OH)D was used, nearly 80% of children failed to reach this threshold.

**TABLE 2:** Groups of vitamin D concentrations stratified by age, season, and gender

Variables	Number	Mean (nmol/L)	*p* value	Vitamin D Status				*p* value
				< 25 nmol/L	25–50 nmol/L	50–75 nmol/L	>75 nmol/L	
				*n* (%)	*n* (%)	*n* (%)	*n* (%)	
Total population	1815	62.29 ± 16.43		25 (1.38)	355 (19.56)	1062 (58.51)	373 (20.55)	
Age group			<.001					<.001
Infant group	120	57.55 ± 27.11		17 (14.17)	21 (17.5)	51 (42.50)	31 (25.83)	
Toddlerhood group	446	67.40 ± 17.03		4 (0.90)	61 (13.68)	235 (52.69)	146 (32.74)	
Preschool group	1249	60.92 ± 14.34		4 (0.32)	273 (21.86)	776 (62.13)	196 (15.69)	
Season			<.001					<.001
Spring	852	64.37 ± 16.87		18 (2.11)	129 (15.14)	493 (57.86)	212 (24.88)	
Summer	187	67.53 ± 18.34		1 (0.53)	28 (14.97)	97 (51.87)	61 (32.62)	
Autumn	651	58.61 ± 14.64		6 (0.92)	169 (25.96)	390 (59.91)	86 (13.21)	
Winter	125	59.39 ± 14.81		0 (0.00)	29 (23.20)	82 (65.60)	14 (11.20)	
Elevation			.049					.016
2–3 km	896	61.76 ± 15.75		7 (0.78)	184 (20.54)	535 (59.71)	170 (18.97)	
3–4 km	848	63.13 ± 16.92		17 (2)	149 (17.57)	492 (58.02)	190 (22.41)	
>4 km	71	58.99 ± 18.41		1 (1.41)	22 (30.99)	35 (49.3)	13 (18.31)	
Gender			.859					<.001
Boy	885	63.99 ± 16.39		12 (1.36)	139 (15.71)	527 (59.55)	207 (23.39)	
Girl	930	60.67 ± 16.31		13 (1.40)	216 (23.23)	535 (57.53)	166 (17.85)	

The Vit E status as shown in Table 3. The deficiency and insufficiency rates were 3.20% and 30.19%. The mean serum tocopherol in the infant group was 8.38 ± 3.25 mg/L, which was higher than that in the toddler (7.50 ± 1.73 mg/L) and preschool groups (7.80 ± 1.41 mg/L). Interestingly, the deficiency rate in the infant group (13.33%) was the highest among the three age groups. The mean serum tocopherol in summer was (8.26 ± 1.96 mg /L), which was higher than that in spring (7.55 ± 1.70 mg /L), autumn (7.86 ± 1.49 mg /L), or winter (7.97 ± 1.82 mg /L). The mean serum tocopherol in the group living above 4 km was (8.01 ± 1.89 mg/L), which was higher than those living at 2–3 km (7.66 ± 1.61 mg/L) and 3–4 km (7.85 ± 1.73 mg/L). One‐way ANOVA showed differences in age, season, and altitude, while the t test showed no difference in serum tocopherol between gender (*p* = .119). Compared with the deficiency rates of Vits A and D (with <50 nmol/L as the standard), the same rates for Vit E in Tibetan children aged 0–6 years in Ganzi Prefecture in China were lower.

**TABLE 3:** Groups of vitamin E concentrations stratified by age, season, and gender

Variables	Number	Mean (mg/L)	*p* value	Vitamin E Status			*p* value
				<5 mg/L	5–7 mg/L	≥7 mg/L	
				*n* (%)	*n* (%)	*n* (%)	
Total population	1815	7.76 ± 1.68		58 (3.20)	548 (30.19)	1209 (66.61)	
Age group			<.001				<.001
Infant group	120	8.38 ± 3.25		16 (13.33)	27 (22.50)	77 (64.17)	
Toddlerhood group	446	7.50 ± 1.73		24 (5.38)	161 (36.10)	261 (58.52)	
Preschool group	1249	7.80 ± 1.41		18 (1.44)	360 (28.82)	871 (69.74)	
Season			<.001				<.001
Spring	852	7.55 ± 1.70		42 (4.93)	284 (33.33)	526 (61.74)	
Summer	187	8.26 ± 1.96		4 (2.14)	42 (22.46)	141 (75.40)	
Autumn	651	7.86 ± 1.49		9 (1.38)	185 (28.42)	457 (70.20)	
Winter	125	7.97 ± 1.82		3 (2.40)	37 (29.60)	85 (68.00)	
Elevation			.024				.573
2–3 km	896	7.66 ± 1.61		32 (3.57)	283 (31.58)	581 (64.84)	
3–4 km	848	7.85 ± 1.73		24 (2.83)	246 (29.01)	578 (68.16)	
>4 km	71	8.01 ± 1.89		2 (2.82)	19 (26.76)	50 (70.42)	
Gender			.119				.091
Boy	885	7.65 ± 1.61		32 (3.62)	285 (32.20)	568 (64.18)	
Girl	930	7.87 ± 1.74		26 (2.80)	263 (28.28)	641 (68.92)	

### 3.2 Factors influencing Vit A, D, and E levels

Polynomial logistic regression was used to analyze the effects of age, season, altitude, and gender on Vit A, D, and E levels, respectively. The results are shown in Table 4. Compared with the normal group, VAD was significantly affected by age, season, and altitude, while MVAD was only significantly affected by season and altitude. According to our calculations of the corresponding *OR* value, it was also found that with increasing altitude, the risk increased over twofold. With increasing age, the risk of VAD was lower compared with spring, and the risk of VAD and MVAD was also reduced in the other seasons. Age, season, and altitude all affected the deficiency and insufficiency of Vit D when compared with the normal group. The *OR* values for the Vit D deficiency and insufficiency groups in autumn and winter were >1, indicating that compared to spring, the incidence of Vit D deficiency and insufficiency increased by approximately twofold. With an increase in age, the incidence of Vit D deficiency decreased, while the incidence of Vit D insufficiency increased. Furthermore, the risk of Vit D deficiency and insufficiency decreased at an elevation of 3–4 km when compared to values seen at 2–3 km, but these differences were not significant at elevations above 4 km. Age and season had an effect on Vit E deficiency and insufficiency when compared to the normal group, but altitude had no significant effect on the findings. As with the results for Vit D, the *OR* value demonstrated that the incidence of Vit E deficiency decreased with age, but the incidence of Vit E insufficiency increased. Furthermore, the incidence of Vit E deficiency and insufficiency was lower in autumn and winter than in spring.

**TABLE 4:** Odds ratios (95% CIs) of VAD, MVAD, vitamin D deficiency, vitamin D insufficient, vitamin E deficiency, and vitamin E insufficient associated with age group

	VAD (<0.70 μmol/L)^a^	MVAD (0.70–1.05 μmol/L)^a^	Vitamin D deficiency (<50 nmol/L)^b^	Vitamin D insufficient (50–74.9 nmol/L)^b^	Vitamin E deficiency (<5 mg/L)^c^	Vitamin E insufficient (5–7 mg/L)^c^
Infant group	1.00	1.00	1.00	1.00	1.00	1.00
Toddlerhood group	0.17 (0.09,0.32)*	0.64 (0.40,1.03)	0.32 (0.18,0.56)*	0.90 (0.55,1.48)	0.43 (0.22,0.87)*	1.71 (1.06,2.78)*
Preschool group	0.24 (0.13,0.42)*	0.90 (0.57,1.40)	0.88 (0.52,1.49)	2.05 (1.27,3.32)*	0.11 (0.05,0.24)*	1.18 (0.75,1.88)
Spring	1.00	1.00	1.00	1.00	1.00	1.00
Summer	0.09 (0.03,0.28)*	0.26 (0.17,0.38)*	0.62 (0.37,1.02)	0.62 (0.43,0.90)*	0.31 (0.11,0.91)*	0.53 (0.36,0.78)*
Autumn	0.72 (0.48,1.07)	0.82 (0.66,1.03)	2.60 (1.84,3.68)*	1.67 (1.25,2.24)*	0.37 (0.18,0.80)*	0.78 (0.62,0.98)*
Winter	0.26 (0.09,0.75)*	0.62 (0.41,0.92)*	2.60 (1.31,5.17)*	2.16 (1.19,3.94)*	0.53 (0.16,1.77)	0.80 (0.52,1.21)
2–3 km	1.00	1.00	1.00	1.00	1.00	1.00
3–4 km	2.88 (1.95,4.25)*	2.00 (1.63,2.46)*	0.72 (0.53,0.98)*	0.78 (0.60,1.00)*	0.67 (0.38,1.17)	0.84 (0.68,1.04)
>4 km	2.92 (1.15,7.46)*	2.93 (1.68,5.10)*	1.34 (0.64,2.82)	0.80 (0.41,1.58)	0.62 (0.14,2.79)	0.73 (0.42,1.26)
Girl	1.00	1.00	1.00	1.00	1.00	1.00
Boy	1.24 (0.86,1.78)	1.02 (0.83,1.24)	0.53 (0.39,0.71)	0.79 (0.62,1.00)	1.43 (0.83,2.46)	1.21 (0.99,1.48)

^a^ The reference group was serum vitamin A ≥ 1.05 μmol/L.

^b^ The reference group was serum 25(OH) D ≥ 75 nmol/L.

^c^ The reference group was serum vitamin E ≥ 7 mg/L.

* *p* < .05.

## 4 DISCUSSION

This study represented a public health physical examination of children. In most urban areas of China, it is recommended by physicians that children have regular health examinations and in particular appropriate Vit A and D supplementation. However, in remote rural areas and ethnic minority areas, this has not been implemented. In this study, 1815 children aged 0–6 in Ganzi Prefecture did not take vitamin supplements regularly.

In this study, there was no significant difference in vitamin levels between genders. Although there is limited evidence of a correlation between vitamin levels and gender, girls have an overall 32% increased risk of vitamin deficiency compared with boys.^30^ Similar studies from South Africa, China, and Ecuador have not found sex‐related differences in vitamin levels.^30^, ^31^, ^32^ Studies have shown that dietary habits and diet structure have a significant impact on metabolism and the status of essential nutrients.^33^ Both Vit A and D firstly increased, but then decreased with age, while Vit E exhibited the opposite effect. Possible explanations for this include: The low Vit A and D levels of neonates at birth may be a normal physiological state that increases with age.^34^ Infant Vit A, and D, is mainly derived from the mother and mother's milk,^35^ whereas Tibetan mothers during pregnancy are given a traditional diet, which is low in Vit A, and D,^36^ such as barley, wheat, potatoes, beef and mutton, yogurt, and butter. During late pregnancy reserves of Vit A, and D are low; therefore, their concentrations in the mother's milk are insufficient to satisfy the needs of the developing fetus, especially normal physiological function and bone formation and they rarely receive foods rich in these vitamins, such as cod liver oil. The serum retinol and 25‐(OH) D levels of Tibetan children in early childhood increased gradually with the addition of dairy products, formula milk powder, nutritional packages, and meat. As preschool Tibetan children grow older, their physical needs increase and their levels of Vit A and D gradually decrease due to their unbalanced diet and dietary structure. Serum tocopherol levels were highest in infancy and lowest in the toddler group, and although they increased gradually with age, they remained lower than the infant levels. This may be associated with breast feeding in infancy, where the Vit E content is higher. With age, the body's demand for Vit E increases, and an unbalanced diet cannot provide sufficient vitamins, resulting in a relatively high level of deficiency.^34^

To determine the relationship between vitamins and seasonal changes, we need to understand that the plateau region of Ganzi Tibet has a monsoon climate, with an altitude of more than 2 km, and its seasonal changes are therefore different from those of the plain regions. Summer is the best season of the year in this region, with relatively sufficient sunshine providing long outdoor activities for children. It is also the harvest season for the Tibetan people, so the children's intake of foods rich in Vits A, D, and E increases significantly. As a result, these vitamin levels in children are highest in summer. At the end of September every year, it begins to snow in this region and can last until the beginning of May in the following year. In spring and summer, the temperature is high; therefore, the children receive more UV exposure, resulting in serum 25‐(OH) D levels being significantly higher in spring and summer than in autumn and winter. However, this study has some limitations. In Ganzi Prefecture, the weather is too cold in winter and spring, and the number of children participating in physical examinations is small.

Based on the changes in Vit A levels at different altitudes, we can see that this is significantly affected by altitude. The higher the altitude, the lower the Vit A level. However, serum 25‐(OH) D levels were not significantly affected by elevation. Although serum tocopherol levels were statistically significant at different elevations, the proportion of these deficiencies was not statistically significant at different elevations.

Daocheng and Xiangcheng counties in Ganzi Prefecture are on the southwest edge of the Sichuan Province, China. Located on the Qinghai‐Tibet Plateau, northeast of the Hengduan Mountains, and has an altitude of 2–4 km. Therefore, due to the influence of altitude, the average temperature, UV levels, and length of sunshine are different. At an altitude of more than 4 km, the temperature is higher and colder, the UV irradiation is stronger, and oxygen partial pressure is reduced, resulting in hypoxia during excess activity. Even in summer and autumn, people often wear thick clothes and hats, resulting in less exposure to outdoor sunshine. Furthermore, living at a higher altitude leads to difficult living conditions because crops are scarce, resulting in worsening economic conditions and therefore a less balanced diet. The diet of children from this region is mainly composed of mantou, Zanba, buttered tea, highland barley, and yogurt, and they also occasionally eat beef and mutton but rarely vegetables, fruit, seafood, pork, or poultry. Therefore, it is very important to use supplements of Vit A, D, and E for these children, especially in the high‐altitude areas.^37^

A growing number of studies have shown that micronutrient deficiencies are significantly related to children's vitamin supplements,^38^ body mass index (BMI)^39^ and diet type.^33^ These data were not included in our study to support the above conclusions. Another major limitation of the present study is that this population was sampled from only one representative area of China. In the future, a large number of multicenter studies are needed. Due to safety and ethical concerns, this study is not a randomized controlled trial, which may cause some bias.

In conclusion, we have found that the Vit A, D, and E levels in children aged 0–6 years in the Tibetan plateau area of Ganzi Prefecture, are related to age, altitude, and season. Because Ganzi Prefecture belongs to the poor areas in China, its economy and culture are relatively backward, there is a lack of knowledge related to health in many of these families and coupled with the unique local eating habits, this results in an overall lower level of healthy children than seen in the lower plain areas. Therefore, a greater distribution of health education is needed to increase nutritional input during pregnancy, maintain a good diet, and promote the use of nutrition packages. Furthermore, children of different ages should be encouraged to modify their outdoor activities according to different altitudes and seasons and to have their nutrition supplemented with preparations of Vit A, D, and E, where necessary, or to consume more food rich in these vitamins. Finally, they should also be exposed to regular physical examinations to prevent the occurrence of diseases associated with vitamin deficiency.

## AUTHOR CONTRIBUTIONS

All authors contributed to the conceptualization, design, and interpretation of the study. Ping Huang, Gang Ke, and Xinmei Lin conceived and designed the study. Ping Huang, Gang Ke, and QW performed the experiments. Wei Lu, Li Zeng, and Shiying Xu analyzed all the data and wrote the article. All authors reviewed and approved the final article.

## FUNDING INFORMATION

The study was supported by Health and family planning research project of Sichuan province (17PJ276)

## CONFLICT OF INTEREST

The authors declare that they have no competing interests.

## Data Availability

The study data can be obtained from the corresponding author on reasonable request.
